# Increased interaction between endoplasmic reticulum and mitochondria following sleep deprivation

**DOI:** 10.1186/s12915-022-01498-7

**Published:** 2023-01-04

**Authors:** Amina Aboufares El Alaoui, Edgar Buhl, Sabrina Galizia, James J. L. Hodge, Luisa de Vivo, Michele Bellesi

**Affiliations:** 1 Department of Experimental and Clinical Medicine, Marche Polytechnic University, Ancona, Italy; 2 School of Biosciences and Veterinary Medicine, University of Camerino, Camerino, Italy; 3 School of Physiology, Pharmacology and Neuroscience, University of Bristol, Bristol, UK; 4 School of Pharmacy, University of Camerino, Camerino, Italy

**Keywords:** Sleep deprivation, Electron microscopy, Neuron, Brain, Mouse, Drosophila

## Abstract

**Background:**

Prolonged cellular activity may overload cell function, leading to high rates of protein synthesis and accumulation of misfolded or unassembled proteins, which cause endoplasmic reticulum (ER) stress and activate the unfolded protein response (UPR) to re-establish normal protein homeostasis. Previous molecular work has demonstrated that sleep deprivation (SD) leads to ER stress in neurons, with a number of ER-specific proteins being upregulated to maintain optimal cellular proteostasis. It is still not clear which cellular processes activated by sleep deprivation lead to ER- stress, but increased cellular metabolism, higher request for protein synthesis, and over production of oxygen radicals have been proposed as potential contributing factors. Here, we investigate the transcriptional and ultrastructural ER and mitochondrial modifications induced by sleep loss.

**Results:**

We used gene expression analysis in mouse forebrains to show that SD was associated with significant transcriptional modifications of genes involved in ER stress but also in ER-mitochondria interaction, calcium homeostasis, and mitochondrial respiratory activity. Using electron microscopy, we also showed that SD was associated with a general increase in the density of ER cisternae in pyramidal neurons of the motor cortex. Moreover, ER cisternae established new contact sites with mitochondria, the so-called mitochondria associated membranes (MAMs), important hubs for molecule shuttling, such as calcium and lipids, and for the modulation of ATP production and redox state. Finally, we demonstrated that Drosophila male mutant flies (elav > linker), in which the number of MAMs had been genetically increased, showed a reduction in the amount and consolidation of sleep without alterations in the homeostatic sleep response to SD.

**Conclusions:**

We provide evidence that sleep loss induces ER stress characterized by increased crosstalk between ER and mitochondria. MAMs formation associated with SD could represent a key phenomenon for the modulation of multiple cellular processes that ensure appropriate responses to increased cell metabolism. In addition, MAMs establishment may play a role in the regulation of sleep under baseline conditions.

**Supplementary Information:**

The online version contains supplementary material available at 10.1186/s12915-022-01498-7.

## Background

Limited energy budget and restricted capability to synthetize new cellular components such as proteins and lipids impose neurons and other cells to function within narrow physiological ranges of activity. Prolonged cellular activity is known to overload cell function and lead to cellular stress [1, 2]. A specific form of cellular stress is the endoplasmic reticulum (ER) stress that can be triggered by high rates of protein synthesis and accumulation of misfolded or unassembled proteins [3]. In normal conditions, cells activate an evolutionary conserved mechanism that tends to re-establish normal protein homeostasis, thus avoiding further damage that could lead to cell death [4, 5]. This homeostatic mechanism, called unfolded protein response (UPR), promotes cellular pathways that degrade misfolded proteins, produces molecular chaperones that limit the aggregation of misfolded proteins, and generally reduces protein synthesis [3, 5].

Neuronal activity changes considerably in response to the animal’s physiological state. Wakefulness heightens metabolism and protein synthesis, whereas sleep reduces them [6]. If wakefulness is prolonged, increased protein demand and sustained metabolism can lead to cellular stress [7, 8]. It has been shown that periods of a few hours of sleep deprivation can induce ER stress in mice leading to changes in the expression of many molecules involved in the UPR [7]. In turn, the expression levels of UPR molecules can modify sleep quantity and quality, contributing for instance to fragmented sleep [9]. The association between UPR and sleep seems particularly critical in aging when the UPR becomes weaker and the possibilities to incur ER stress and cell damage becomes elevated [10].

The ER is a large, continuous membrane system, whose size and morphology can change considerably in response to specific demand [11, 12]. The different ER morphologies can be associated with distinct cellular states. For example, ER stress has been linked to significant expansion of ER cisternae through the generation of new ER sheets [13]. Of particular importance are the contact sites that the ER develops with other subcellular organelles and/or with the plasma membrane (PM). Such interactions are essential for regulating intracellular signaling, including calcium and lipid trafficking between one cellular compartment to another one [14–16]. Under ER stress conditions, stress signals are delivered to mitochondria via specific ER–mitochondria contact sites, also known as mitochondria-associated membranes (MAMs). These interaction sites also play an important role in modulating mitochondrial shape and motility and are significant hot spots for controlling and coordinating the cellular response to stress [17, 18].

In this study, we combined gene expression analysis and electron microscopy to determine whether the molecular fingerprints of ER stress induced by acute sleep deprivation are associated with structural ER modifications and with the formation of MAMs or contact sites with PM. In addition, we employed a transgenic *Drosophila* model with increased ER-mitochondria interactions to causally force their interactions and measure the effect on sleep and wake behavior.

## Results

### Sleep deprivation upregulates genes involved in ER stress response, MAMs, and mitochondrial activity

To identify specific ER and mitochondria genes that are differentially expressed in sleep deprivation (SD) relative to undisturbed sleep (S), we interrogated a gene dataset that was previously used to detect genes modulated by sleep and wake in oligodendrocytes versus other brain cells (mainly neurons and astrocytes) [19]. In the present study, we used the array data from the non-oligodendrocyte cells, obtained from the forebrain of mice sacrificed after 6–7 h of sleep during the light phase (S, *n* = 6) and mice sleep deprived during the day via exposure to novel objects (SD, 4 h of forced enriched wake during the light phase, *n* = 6).

**Fig. 1:**
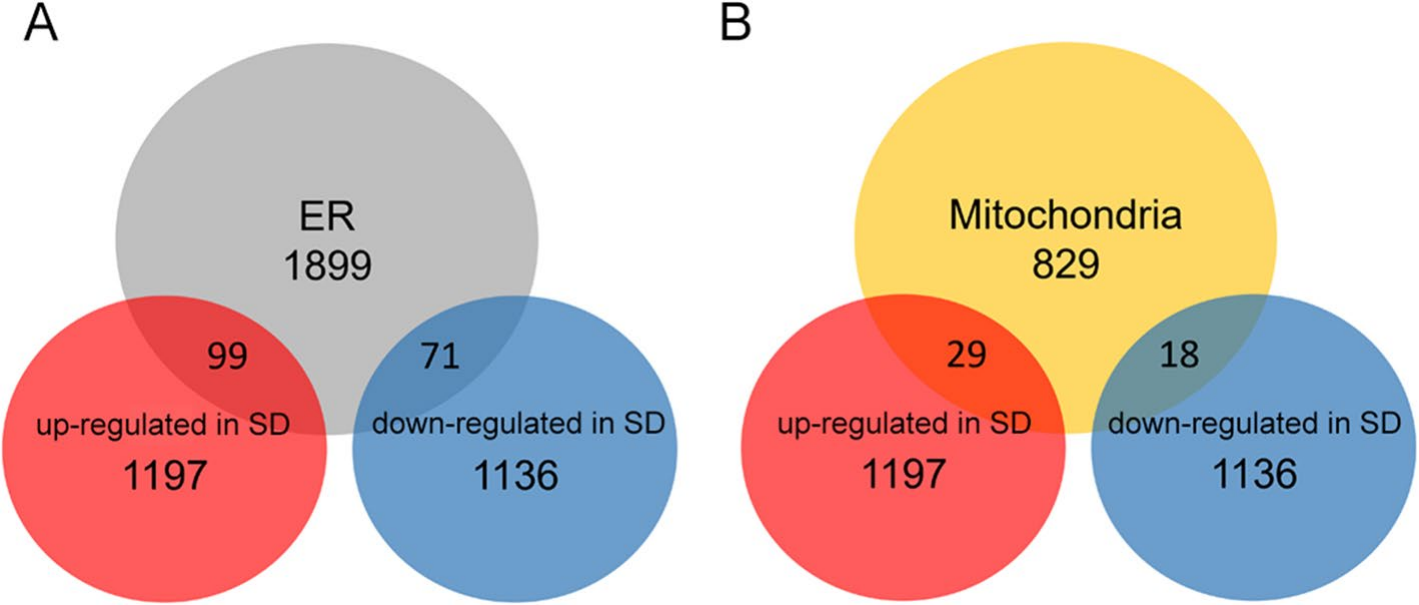
**A**, **B** Venn diagrams showing the number of ER (**A**) and mitochondrial (**B**) genes upregulated and downregulated in SD

**Fig. 2:**
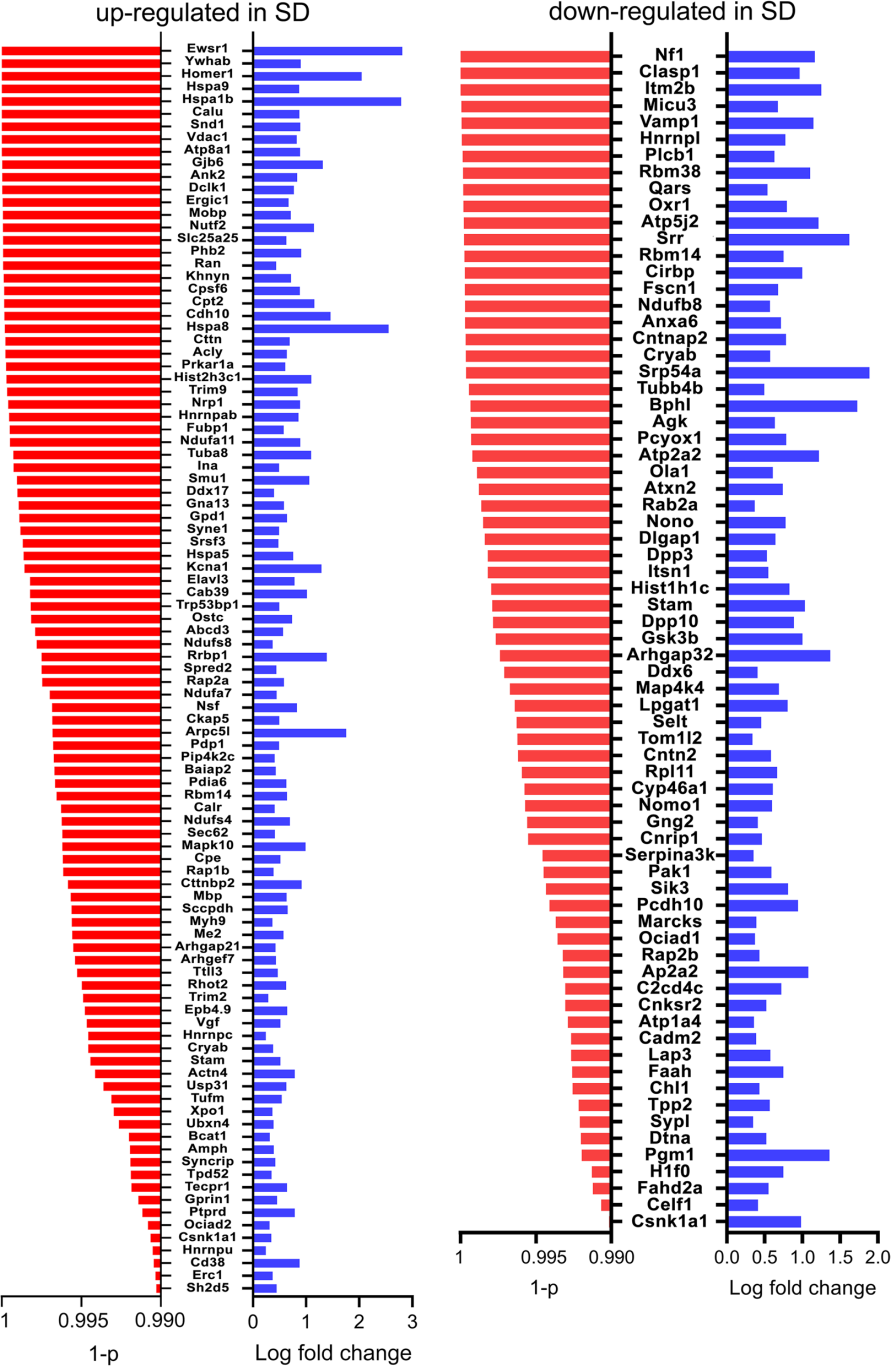
ER genes upregulated and downregulated in SD are ranked in order of significance (red bars, Welch’s *t* test with Benjamini and Hochberg FDR multiple-test correction). The log fold change is represented by the blue bars

**Fig. 3:**
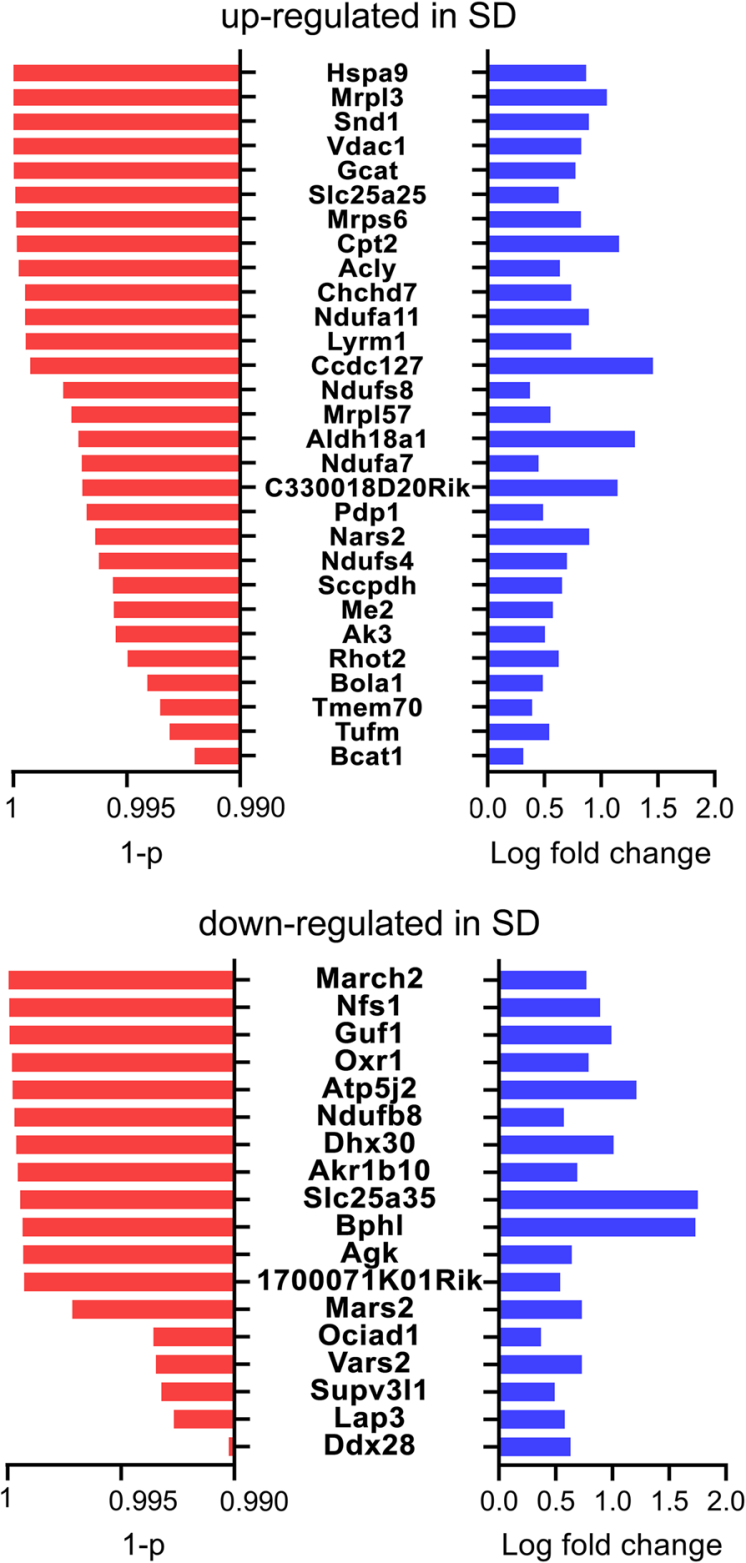
Mitochondrial genes upregulated and downregulated in SD are ranked in order of significance (red bars, Welch’s *t* test with Benjamini and Hochberg FDR multiple-test correction). The log fold change is represented by the blue bars

Using an FDR of 1%, we identified 2538 genes that changed their expression because of SD. Of those, 1341 genes were upregulated while 1197 gene were downregulated after SD. Of note, we found that Hspa9 and Vdac1 were among the highly significant ER and mitochondrial genes overexpressed after SD, suggesting an important response of the ER and mitochondria to SD. Hence, we focused the analysis on ER and mitochondria by intersecting upregulated and downregulated SD gene lists with the mouse brain ER (ER genes containing 1899 unique genes) and mitochondrial (Mito genes containing 829 unique genes) gene lists to finally identify the genes that were differentially expressed in SD and that also coded for proteins enriched in these organelles. The resulting Venn diagrams showed that among the ER genes, 99 genes were upregulated after SD (ER-SDup) while 71 genes were downregulated (ER-SDdown). In addition, among the Mito genes, 29 genes were upregulated after SD (Mito-SDup) while 18 genes were downregulated after SD (Mito-SDdown, Figs. 1, 2, and 3).

To identify which cellular processes might be affected by the upregulation and downregulation of the different sets of genes after SD, we performed a protein-protein interaction (PPI) network analysis that revealed that upregulated SD genes were associated with significantly different transcriptionally reshaped landscapes (nodes) for ER unfolding protein response and chaperone function, and respiratory chain activity. Both ER-SDup and Mito-SDup networks had significantly more interactions than chance (ER-SDup: number of nodes: 99; number of edges: 132; average node degree: 2.67; average local clustering coefficient: 0.37, PPI enrichment *p*-value: 2.81e−12. Mito-SDup: number of nodes: 29; number of edges: 22; average node degree: 1.52; average local clustering coefficient: 0.36, PPI enrichment *p*-value: 5.16e−08). By contrast, downregulated SD genes were associated with significantly different clusters mainly involving mitochondrial processes, such as mitochondrial ribosomal subunit assembly and aminoacyl-tRNA biosynthesis (Mito-SDdown: number of nodes: 18; number of edges: 7; average node degree: 0.8; average local clustering coefficient: 0.3, PPI enrichment *p*-value: 3.04e−05), whereas the ER-SDdown network analysis showed no significant cluster (number of nodes: 71; number of edges: 29; average node degree: 0.8; average local clustering coefficient: 0.44, PPI enrichment *p*-value: 0.09 (Table 1; Additional file 1: Figure S1-4).

**Table 1:** Functional enrichment of ER genes upregulated in SD and Mito genes upregulated and downregulated in SD (top 10 biological processes with strength of enrichment > 1)

#Term ID	Term description (biological process)	Strength	False discovery rate	Observed gene count	Background gene count
**ER genes upregulated in SD**					
GO:0042026	Protein refolding	1.77	0.00056	4	15
GO:0006611	Protein export from nucleus	1.19	0.0013	6	87
GO:0006986	Response to unfolded protein	1.11	0.0027	6	104
GO:0051085	Chaperone cofactor-dependent protein refolding	1.49	0.0034	4	29
GO:0031532	Actin cytoskeleton reorganization	1.21	0.0049	5	69
GO:0034620	Cellular response to unfolded protein	1.18	0.0061	5	74
GO:0071320	Cellular response to camp	1.27	0.0132	4	48
GO:1900024	Regulation of substrate adhesion-dependent cell spreading	1.23	0.0183	4	53
GO:0098974	Postsynaptic actin cytoskeleton organization	1.48	0.0259	3	22
GO:0070934	CRD-mediated mRNA stabilization	1.95	0.0463	2	5
**Mito genes upregulated in SD**					
GO:0032543	Mitochondrial translation	1.75	0.0034	4	54
GO:0043604	Amide biosynthetic process	1.09	0.0034	7	434
GO:0006412	Translation	1.16	0.0053	6	316
GO:0045333	Cellular respiration	1.36	0.0241	4	133
GO:0032981	Mitochondrial respiratory chain complex i assembly	1.7	0.0273	3	46
GO:0046034	ATP metabolic process	1.25	0.0473	4	172
**ER genes downregulated in SD**					
GO:1902775	Mitochondrial large ribosomal subunit assembly	2.91	0.0031	2	3
GO:0043604	Amide biosynthetic process	1.07	0.0317	4	414
GO:0007005	Mitochondrion organization	1.1	0.0317	4	388
GO:0006418	tRNA aminoacylation for protein translation	1.76	0.0348	2	43

Moreover, we carried out a PPI network analysis on the ER and mitochondrial genes that were not affected by SD to potentially reveal biological processes not influenced by SD. Considering the high number of transcripts involved, we found 1629 nodes with 29718 edges (average node degree: 36.5 average local clustering coefficient: 0.338, PPI enrichment *p*-value: < 1.0e−16) for ER and 748 nodes with 16539 edges (average node degree: 44.2 avg. local clustering coefficient: 0.415, expected number of edges: 2282, PPI enrichment *p*-value: < 1.0e−16) for mitochondrial genes. Highly enriched and mostly represented biological processes related to ion transport across membranes and acetyl-CoA biosynthetic process from pyruvate. A summary of these results is reported on Additional file 2 and 3.

**Fig. 4:**
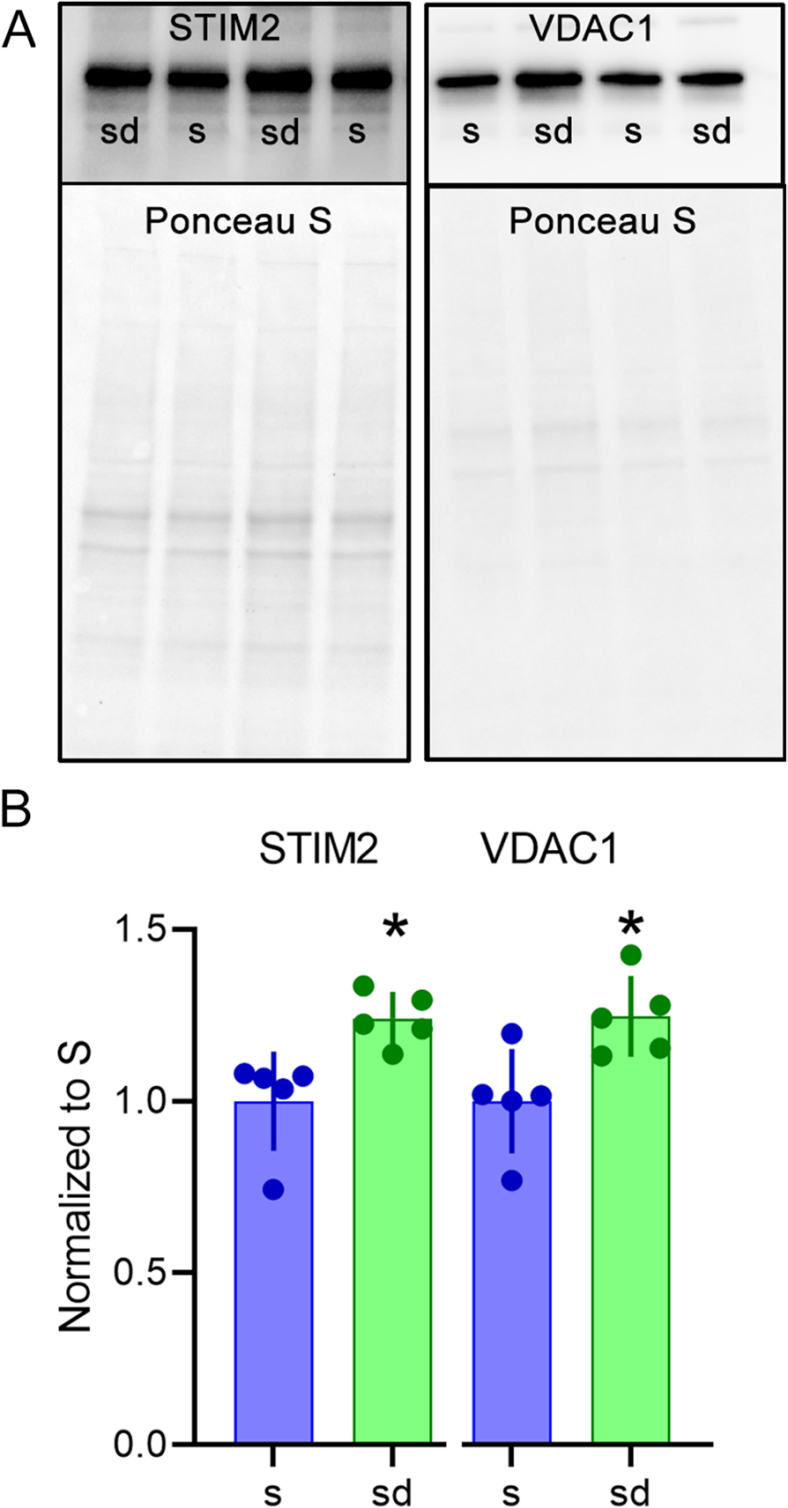
Western blot analysis for VDAC1 and STIM2. **A** VDAC1 and STIM2 bands in two *S* and *SD* representative samples (upper panel) and their relative bands showing total protein staining with Ponceau S (lower panel). **B** Semiquantitative analysis of their expression relative to sleep (*S*, *n* = 5; *SD*, *n* = 5). Bars indicate mean values ± standard deviation (std); dots indicate single values in this and all subsequent figures. Mann-Whitney test, *p < 0.05

To confirm that differentially expressed transcripts were indeed associated with protein changes, we measured the expression of two proteins whose mRNA transcripts values were significantly upregulated in SD relative to S. We analyzed the levels of VDAC1 and STIM2 in brain homogenates of S and SD mice by western blotting. VDAC1 is a protein participating in the formation of MAMs, while STIM2 contributes to calcium uptake from the extracellular environment [20, 21]. Semiquantitative analysis at western blotting confirmed that VDAC1 and STIM2 levels were higher in SD than S (VDAC: +24.7%, *p* = 0.03; STIM2: +24.1%, *p* = 0.008, Fig. 4, Additional file 4 and 5).

### Sleep deprivation increases ER-mitochondrial interaction

**Fig. 5:**
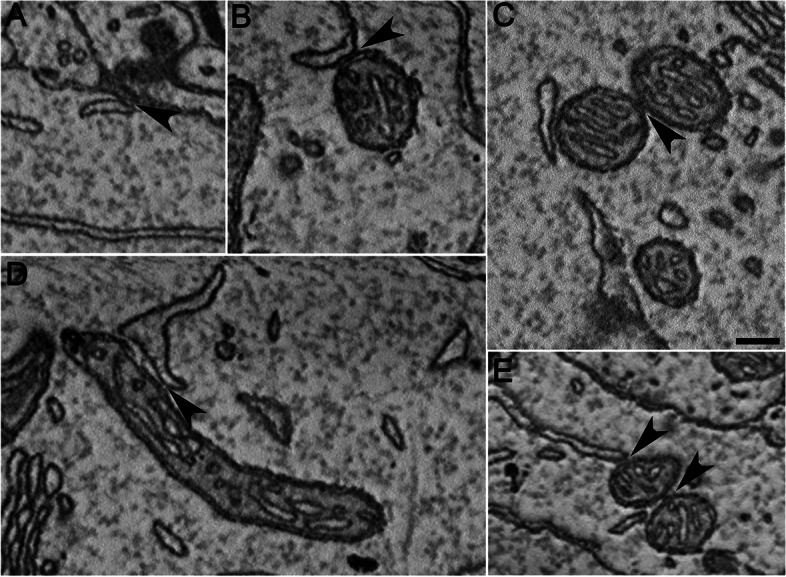
Electron micrographs showing examples of contact sites between ER cisternae and plasma membrane (PAMs, **A**), ER and mitochondria (MAMs, **B**–**E**), and among mitochondria (**C**, **E**). Arrows indicate the contact site (apposition < 20 nm). Scale bar: 200nm

Transcriptomic analysis revealed that ER and mitochondria strongly respond to SD with changes in the expression of genes involved in specific functions, such as ER stress, MAMs, calcium trafficking, and mitochondrial respiratory activity. To evaluate whether these SD dependent molecular effects were associated with ER and mitochondrial structural modifications, we analyzed electron microphotographs of 139 and 132 neurons from the frontal motor cortex of S and SD mice, respectively. We focused on neuronal somas where there is abundance of ER cisternae and mitochondria (Fig. 5).

**Fig. 6:**
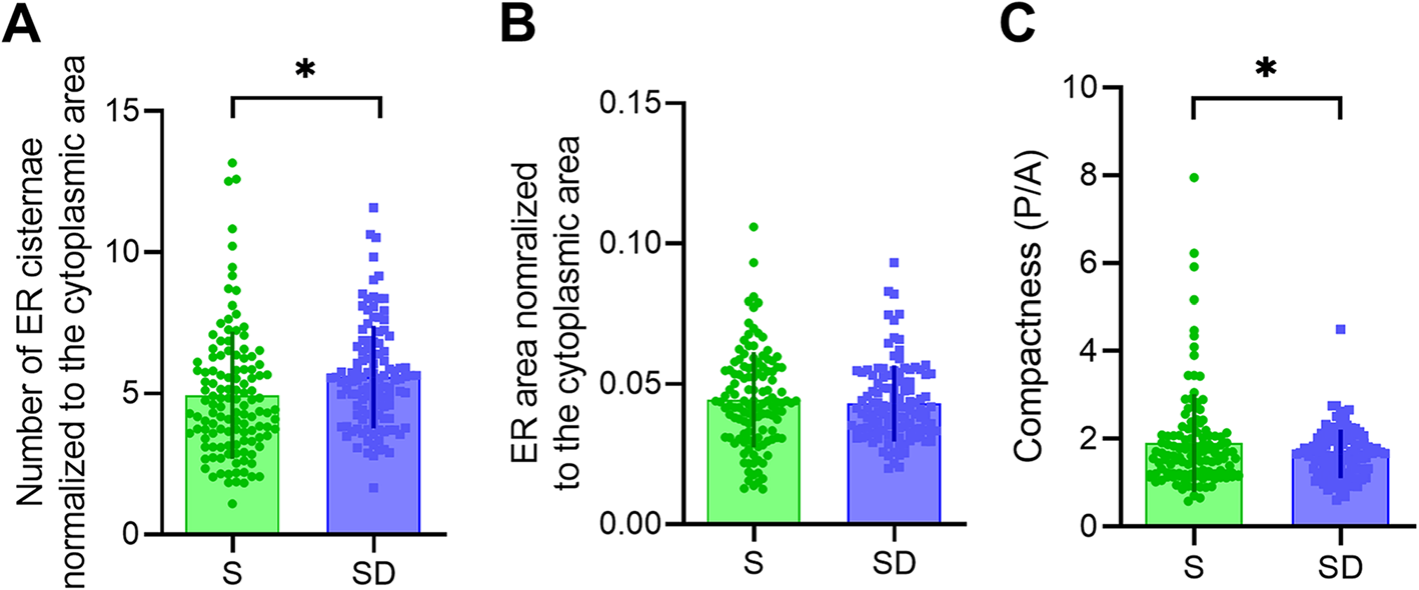
ER ultrastructural morphology in S (green) and SD (blue). **A**, **B** Quantification of number (**A**) and area (**B**) of ER cisternae normalized to the cytoplasmic area. **C** Measure of compactness of the ER cisternae estimated as the ratio between the ER perimeter and area. Unpaired *t*-test (*S*, *n* = 139; *SD*, *n* = 132) *p < 0.05

**Fig. 7:**
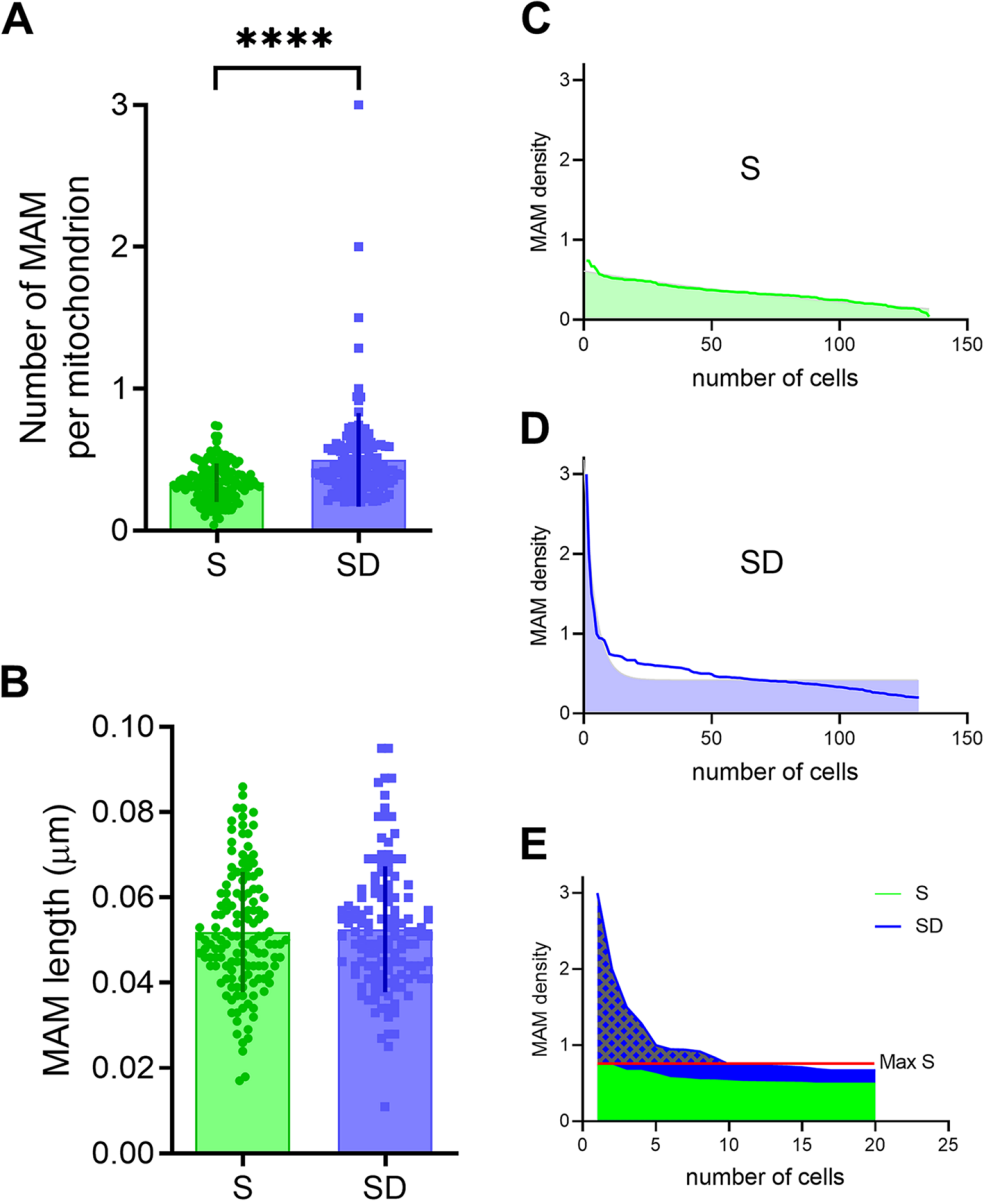
MAM characterization in S and SD. **A**, **B** Quantification of MAMs number (**A**) and lengths (**B**) in S (green) and SD (blue). Unpaired *t*-test (*S*, *n* = 139; *SD*, *n* = 132) ****p < 0.0001. **C**–**E** Distribution of MAMs in S (**C**) and SD (**D**) ranked by density; lines are representing the fitting curves of the distribution. **E** Distributions overlap for *S* (green) and *SD* (blue) for the top 20 cells highlighting remarkable high differences in MAM density. Note the presence of very high MAM density (dashed pattern above the highest *S* value, red line) in 10 SD cells

Manual segmentation and analysis of ER and mitochondria showed that the density of ER cisternae (number of ER cisternae normalized by cytoplasmic area) was higher in SD relative to S (+13.1 ± 0.67%, *p* = 0.015), while the overall area occupied by ER cisternae did not change (*p* = 0.52). Although the ER area was not affected by SD, ER bidimensional shape changed in SD. By estimating the ER compactness (i.e., a measure representing the degree to which a shape is compact) through the measure of perimeter to area ratio, we found that ER shape in SD was less convoluted and more rounded than in S (*p* = 0.03, Fig. 6). Next, we focused on the MAMs, points of interaction between ER and mitochondria, and we quantified their number and length in S and SD. We found that, while the average length of MAMs did not differ between S and SD (*p* = 0.7), the ER in the SD condition had about 50% more contacts with mitochondria (higher number of MAMs per mitochondrion) relative to S (+ 47.8 ± 3.2%, *p* < 0.0001). Interestingly, a small portion of cells (~10) out of more than 100 showed very high values of MAMs density (up to two-three times the values observed in S, Fig. 7).

**Fig. 8:**
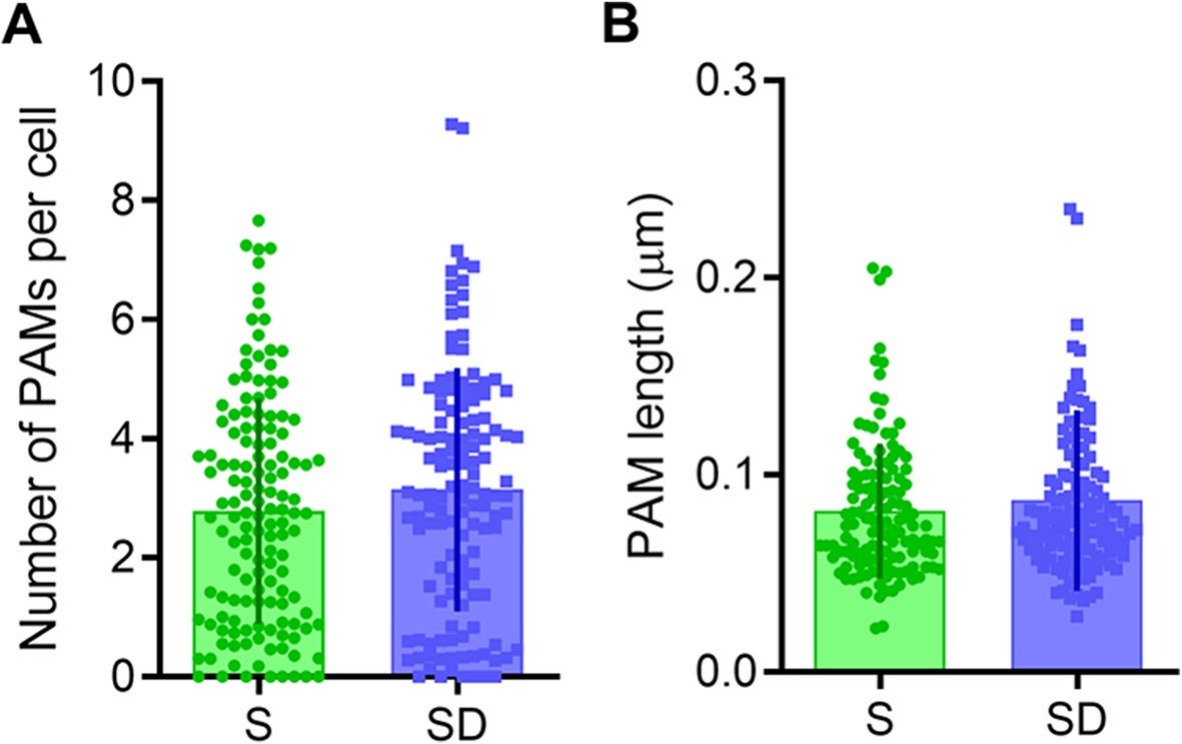
**A**, **B** Quantification of number (**A**) and length (**B**) of contact sites between ER and plasma membrane (PAMs) in *S* (green) and *SD* (blue). Unpaired *t*-test not significant (*S*, *n* = 139; *SD*, *n* = 132)

Using the same criteria adopted for MAM detection, we also studied the points of contacts between the ER and plasma membrane (called PAMs), finding that both PAM length and number did not change between S and SD (length: *p* = 0.29; number: *p* = 0.13, Fig. 8).

**Fig. 9:**
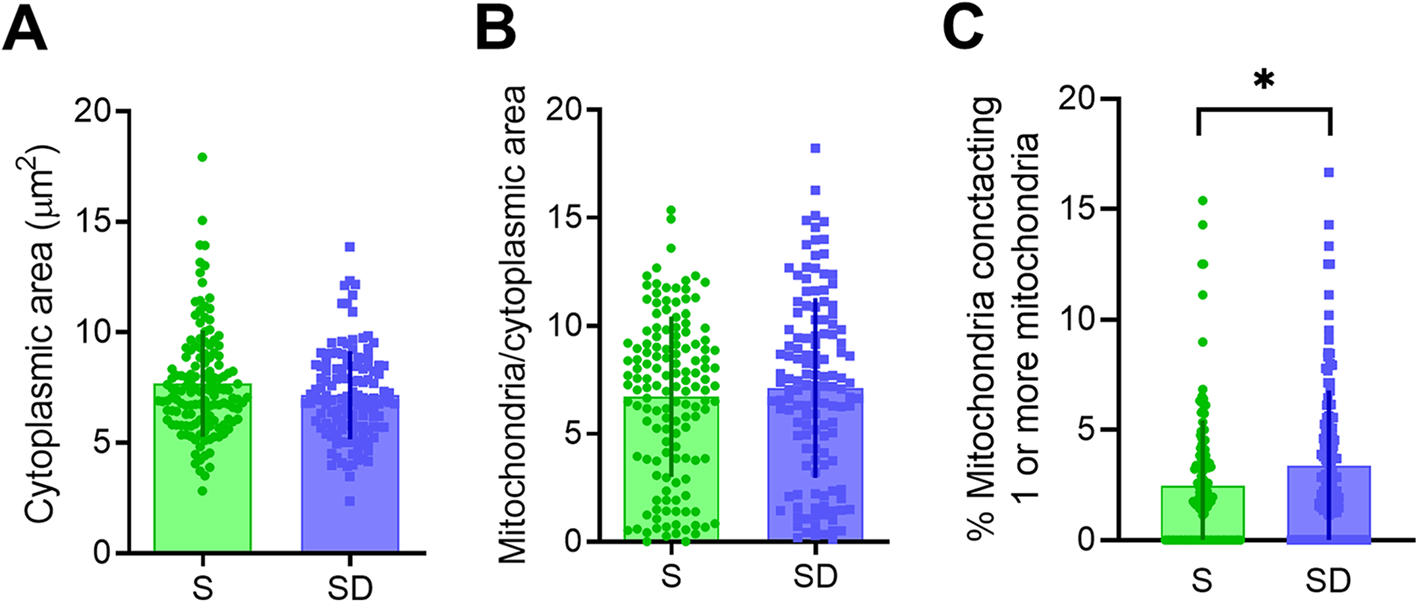
Mitochondrial ultrastructural evaluation. **A**–**C** Quantification of cytoplasmic area (**A**), mitochondrial size (**B**), and contacts between them (**C**) in *S* (green) and *SD* (blue). Unpaired *t*-test (*S*, *n* = 139; *SD*, *n* = 132) *p < 0.05

Finally, we assessed morphological changes in mitochondria by measuring the density of mitochondria, their areas, and the number of contacts they made with each other. Neither density nor mitochondrial area were affected by SD (density: *p* = 0.82; area: *p* = 0.41). However, we found that SD was associated with a higher number of contacts between mitochondria, suggestive of an increased necessity for mitochondria to form clusters (+ 36.4 ± 4.9%, *p* = 0.02, Fig. 9). Finally, we quantified the number of contacts between mitochondria and plasma membrane, finding that this occurrence was more frequent in SD than S (*p* = 0.048), although it was overall very rare in both conditions (~0.01 per cell, not shown).

### Induction of artificial ER-mitochondrial interactions reduces sleep time

To clarify whether MAMs formation played some active role in regulating sleep, we took advantage of a *Drosophila* model, in which the formation of MAMs was forced by the overexpression of a targetable synthetic linker between the mitochondria and the ER in brain neurons. Male mutant flies (*n* = 181, *elav > linker*) and both parental controls (*n* = 118, *elav/+*, *n* = 212, *linker/+*) were housed in monitor chambers for 4 consecutive days to obtain a reliable measure of their sleep and wake activity.

**Fig. 10:**
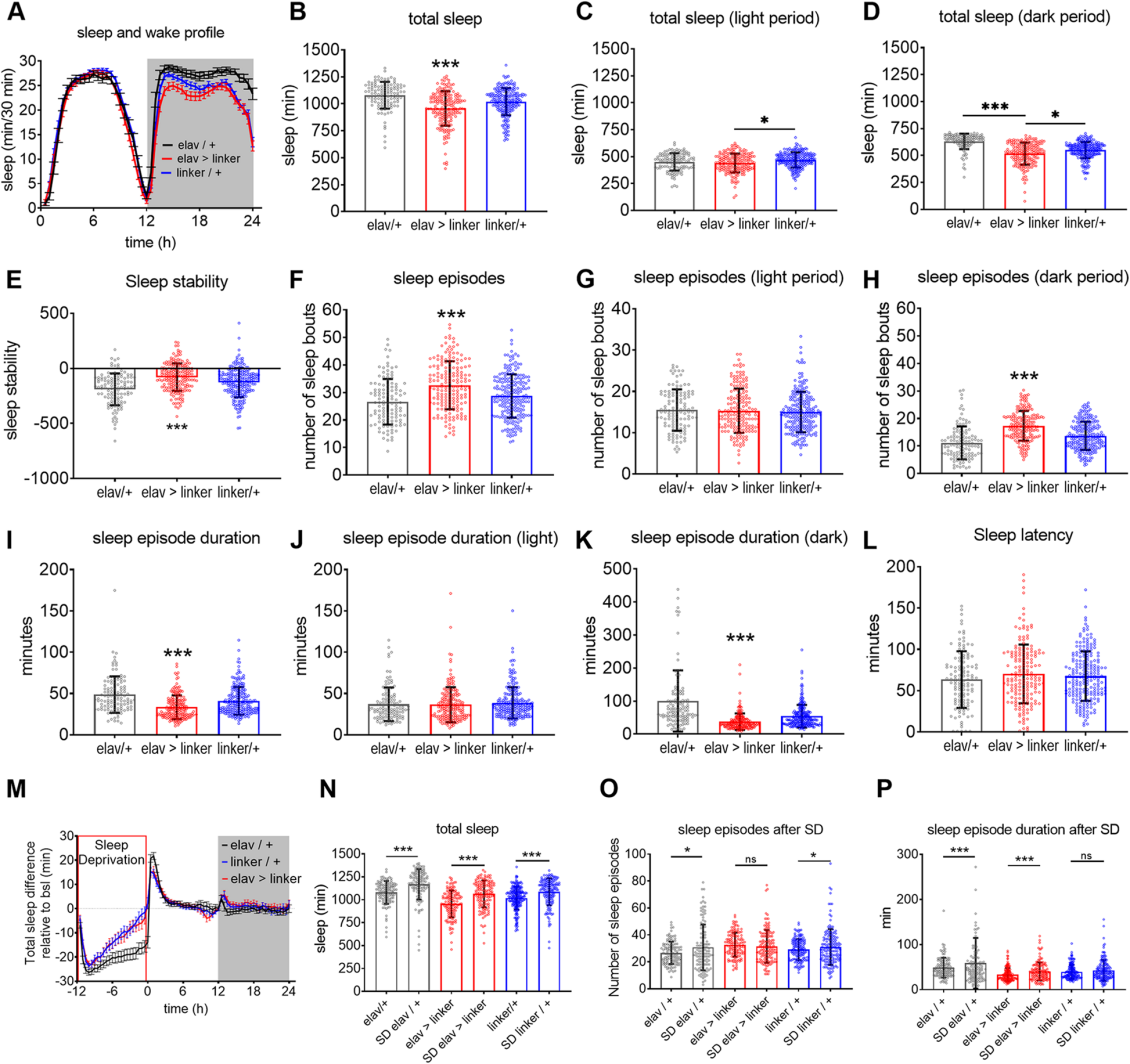
Sleep and sleep deprivation in *elav > linker* (red, *n* = 181) flies and relative controls [*elav/+* (black, *n* = 118) and *linker/+* (blue, *n* = 212)]. **A** 24 h sleep profile, plotted as time asleep within 30 min bins, over the day (grey, lights off; white, lights on). **B**–**D** Total sleep quantification over the 24 h (**B**), during light (**C**) and dark (**D**) periods. **E** Sleep stability calculated as maximum sleep minus maximum wake duration. **F**–**H** Number of sleep episodes over the 24 h (**F**), during light (**G**) and dark (**H**) periods. **I**–**K** Mean duration of sleep episodes over the 24 h (**I**), during light (**J**) and dark (**K**) periods. **L** Sleep latency. Kruskal-Wallis followed by Dunn’s multiple comparisons, ***p < 0.001. **M** Sleep profile after 12 h of SD. **N** Total sleep quantification over the 24 h baseline (BSL) and for the day after SD. **O** Number of sleep episodes over the 24 h BSL and after SD. **P** Duration of sleep episodes over the 24 h BSL and after SD. Kruskal-Wallis’s test for genotype effect, Wilcoxon’s test for pair-wise comparisons, *p < 0.05, ***p < 0.001

Overall, linker-mutant flies slept less than both wild type controls (*elav > linker vs elav/+*: − 11.5 ± 0.18%, *p* < 0.001; *elav > linker vs linker/+*: − 6.2 ± 0.09%, *p* < 0.001). This decrease was particularly evident during the dark period (*elav > linker vs elav/+*: − 18 ± 0.32%, *p* < 0.001; *elav > linker vs linker/+*: − 6.2 ± 0.11%, *p* < 0.001), when flies are usually mostly asleep. The reduction in sleep time was accompanied by an overall increased number of sleep episodes of shorter duration (*number*: *elav > linker vs elav/+* : + 22.6 ± 0.79%, *p* < 0.001; *elav > linker vs linker/+*: + 13.7 ± 0.38%, *p* = < 0.001; *duration*: *elav > linker vs elav/+*: − 31.3 ± 1.64%, *p* < 0.001; *elav > linker vs linker/+*: − 18.5 ± 0.79%, *p* < 0.001) culminating in a decreased sleep stability (*elav > linker vs elav/+*: − 58 ± 7.85%, *p* < 0.001; *elav > linker vs linker/+*: − 38.3 ± 5.18%, *p* = 0.001). Sleep latency did not differ among groups (elav/+ *vs* elav > linker: *p* = 0.27; *elav > linker vs linker/+*: *p* = 0.99). When challenged with 12 h of enforced wake during the dark period, all groups of flies responded with increased sleep duration the following day (*elav/+ n* = 118, *elav > linker n* = 139, *linker/+ n* = 155; *elav/+* SD *vs elav/+*: + 8.2 ± 0.14%, *p* < 0.001; *elav > linker* SD *vs elav > linker*: + 11.4 ± 0.2%, *p* < 0.001; *linker/+* SD *vs linker/+*: + 8.4 ± 0.12%, *p* < 0.001). No effect of genotype was found in the amount of sleep rebound (*p* = 0.38). Analysis of number and mean duration of sleep episodes did not reveal substantial differences of mutant flies relative to controls in response to SD (*number*: *elav/+* SD *vs elav/+*: + 15.3 ± 0.9%, *p* < 0.001; *elav > linker* SD *vs elav > linker*: − 4 ± 0.16%, *p* = 0.3; *linker/+* SD *vs linker/+*: + 6.6 ± 0.27%, *p* = 0.12; *duration elav/+* SD *vs elav/+*: + 58 ± 5.9%, *p* < 0.001; *elav > linker* SD *vs elav > linker*: +20.9 ± 1.17%, *p* = 0.0011; *linker/+* SD *vs linker/+*: + 8.3 ± 0.44%, *p* = 0.14, Fig. 10).

Thus, enhancing the formation of MAMs in fly brain neurons promoted shorter and less consolidated sleep, while the response to SD remained unchanged.

## Discussion

Here, we show that SD is associated with transcriptional modifications of genes involved in ER stress, MAMs formation, and mitochondrial activity. We also show that the number of contacts that mitochondria establish with ER cisternae and with other mitochondria and increases after SD. Moreover, we demonstrate that forcing the formation of MAMs in flies reduces the amount and the consolidation of sleep, without altering the homeostatic response to SD.

MAMs are the ER regions that mediate communication between the ER and mitochondria [22, 23]. They have a specific structure and form a microdomain enriched in proteins and lipids involved in distinct signaling functions, which include control and regulation of cellular homeostasis and survival, metabolism, and apoptosis [18]. Morphologically, they are characterized by an apposition between the ER and mitochondria at distances of approximately 10–25 nm [24]. In SD animals, we found that the number of MAMs in cortical neurons is increased by almost 50% relative to sleep. This change could be a compensatory mechanism aimed at enhancing molecule shuttling between the ER and the mitochondria, in response to the increased metabolic activity that characterizes even short periods of extended wake.

Lipids are among the molecules that require continuous exchange between mitochondria and ER. The ER represents the major “lipid factory” within the cell, being capable of synthesizing structural phospholipids, sterols, and energy storage lipids, such as triacylglycerols and steryl esters [25, 26]. The ER supplies a large portion of these lipids to other organelles via vesicles or by lipid transfer proteins at membrane contact sites such as MAMs [27]. MAMs are enriched with proteins involved in the synthesis and transport of important phospholipids, such as phosphatidylserine and phosphatidylcholine, which are among the main components of cellular membranes and exert important functions inside and out of mitochondria [28, 29]. For instance, it has been shown that in two *Drosophila* models of Parkinson’s disease (loss-of-function mutations in *parkin* and *pink1*) an increased number of MAMs and an altered lipid trafficking towards mitochondria can lead to phosphatidylserine depletion from the ER [30]. Loss of phosphatidylserine caused a defect in the production of neuropeptide-containing vesicles that controlled circadian rhythms and sleep patterns. Notably, the sleep disturbances were reversed by feeding *parkin* and *pink1* fly mutants with a phosphatidylserine enriched diet that rescued the neuropeptidergic vesicle production. Hence, increased number of MAMs following SD not only could reflect the necessity to enhance phospholipid production rates in neurons but might also lead to impaired synaptic communication if neurotransmitter vesicle production by the ER is affected.

Calcium is also frequently shuttled through MAMs: calcium is released from the ER to mitochondria where it is necessary for regulating the bioenergetics of the cell, as the enzymes implicated in energy production (e.g., in the tricarboxylic acid cycle and the electron transport chain) largely depend on calcium to generate ATP [31]. Upon cellular activation, calcium request can further increase. A few hours of SD increase the production of cytochrome oxidase components and boosts the activity of the mitochondrial respiratory chain, in the effort of generating more ATP [32]. In support to the hypothesis that SD enhances calcium transport from ER to mitochondria, many of the transcripts that we found to be upregulated in SD mice are actively implicated in calcium homeostasis and shuttling. Among them, we can mention Hspa9, Vdac1, and Stim2. The Hspa9 gene is located on chromosome 5q31.2 and encodes for one of the heat shock protein 70 family members, also known as GRP75 (glucose-regulated protein 75). This protein functions as a molecular chaperone and regulates cellular stress response, cell proliferation, and apoptosis by interacting with other proteins [33]. It has been found in the mitochondria, endoplasmic reticulum, and plasma membrane, though it is present in other subcellular compartments. GPR75 ties the N-terminal domain of the ER Ca2+ channel inositol 1,4,5-trisphosphate receptor (IP3R) to the voltage-dependent anion-selective channel protein 1 (VDAC1), creating a molecular bridge that increases Ca^2+^ transfer from ER to mitochondria by stabilizing the coupling of the two receptors [28]. The IP3R-GRP75-VDAC complex is enriched, though not exclusively present, at the MAMs, where it plays a crucial role in the regulation of the intracellular Ca^2+^ concentration [29]. The Vdac1 gene is located on the chromosome 5q31.1 and encodes for the protein VDAC1 (voltage-dependent anion-selective channel 1) that forms an ion channel in the outer mitochondrial membrane (OMM) where regulates the crosstalk between mitochondria and the rest of the cell. Specifically, VDAC1 mediates both the Ca^2+^ transport from the ER to mitochondria at MAMs and the cellular energy production by transporting ATP/ADP, NAD+/NADH and acyl-CoA from the cytosol to the intermembrane space. Moreover, it is responsible for the metabolite and ion transport and takes part in apoptosis [34]. Stim2 codes for a protein that belongs to the stromal interaction molecule (STIM) family. Reduction or depletion of Ca^2+^ in the lumen of the ER triggers STIM2-mediated activation of plasma membrane Orai Ca^2+^entry channels which increases calcium influx into the cytosol followed by replenishment of the ER by the sarco-endoplasmic reticulum Ca^2+^-ATPase (SERCA) [35].

Another gene that is linked to Ca^2+^ dynamics and is upregulated by SD is slc25a25 that encodes for the protein SLC25A25 (Ca^2+^-binding mitochondrial carrier protein SCaMC-2) and has a significative expression in mitochondria. This protein is present in the inner membrane of mitochondria where functions as shuttle for metabolites, nucleotides, and cofactors between the cytosol and mitochondria and as ATP-Mg2^+^/Pi carrier catalyzing an electroneutral exchange of ATP- Mg2^+^ for Pi [36].

Increased Ca^2+^ shuttling from ER to mitochondria helps ATP generation, which is necessary to produce more ER chaperones and guarantee proper protein folding [37, 38]. SD is commonly associated with the accumulation of misfolded proteins and ER stress that triggers the UPR [7, 39]. The increased need for ATP production would also explain why mitochondria tend to form clusters in the SD condition. The observed clustered mitochondria could represent organelle tethering, the first step in the fusion process. In general, during elevated levels of cellular stress, mitochondrial fusion promotes the formation of elongated mitochondrial networks that facilitates the distribution of the matrix components and the stimulation of respiratory activity [40, 41]. Furthermore, mitochondrial clustering relates to cell survival mechanisms and protects mitochondria from autophagic degradation, by maximizing ATP synthase activity and maintaining ATP production, supplying autophagosomal membranes during starvation [42].

Cellular stress is often accompanied by imbalanced redox state and more abundant reactive oxygen species (ROS) that can damage many cellular components. Oxidative stress can lead to the formation of stress granules (SGs), membraneless ribonucleoproteins containing mRNA being stopped at the initiation of translation [43]. Along this line, we found increased expression of snd1 in association with sleep deprivation. Snd1 gene codes for the protein Staphylococcal Nuclease Domain-containing protein 1 (SND1), also known as Tudor-SN or p100 which is a multidomain protein involved in the formation of SGs. Together with other stress granule proteins, SND1 helps stabilizing mRNA, degrading highly mutated, hyper-edited regions of double stranded RNAs generated during the cellular stress response [44]. The increased expression of snd1 in response to acute SD could be protective against ROS, ER stress, and have an antiapoptotic role [43, 45]. ROS could also participate in the regulation of sleep homeostasis. Recent research showed that numerous short-sleeping *Drosophila* mutants are susceptible to oxidative stress, exhibiting shorter survival times than controls, and that increasing sleep via genetic or pharmacological manipulations reduces susceptibility to oxidative stress [46]. In addition, boosting the expression of antioxidant enzymes suppressed daily sleep in flies. The mechanism linking cellular ROS concentration to sleep homeostasis has not been completely defined yet, but Kempf and colleagues recently demonstrated that Kvβ subunit (Hyperkinetic; Hk) of the potassium ion channel Shaker can act as a redox sensor that increases its activity in response to ROS production in the sleep promoting neurons of the fly brain. The oxidation of the cofactor associated with the shaker channel would decrease potassium conductance, depolarize the membrane potential, and boost the frequency of action potentials in these neurons, thereby promoting sleep [47].

All together these findings suggest that wake-induced ER stress, MAM formation, increased mitochondrial activity, calcium homeostasis, and ROS production are all linked mechanisms that may play a role in sleep homeostasis. They could therefore represent the mechanisms underpinning the building up of sleep pressure at the cellular level. To this regard, it has been proposed that ER stress and the UPR could be related to local sleep, a form of cellular quiescence induced by previous intense activity that appears as a brief period of neuronal silence (OFF period) at the level of extracellular cortical electrophysiological recordings [48]. Local sleep is thought to affect isolated cell assemblies that build up enough “tiredness” or “fatigue” and then require periods of cellular rest, which translate into reduced synaptic activity and local electrical silences [49]. Interestingly, we found that the increase in MAMs density in cortical neurons after SD was not homogeneous. Indeed, about 10% of neurons displayed remarkably high densities of MAMs. Thus, it is possible that during SD some neurons tend to fatigue more and display higher levels of ER stress than others. This would be compatible with the fact that only some groups of neurons engage in a local sleep state during SD. Thus, thanks to local sleep, neurons could re-establish cellular homeostasis and alleviate the burden of wake-related activity. The underlying mechanism is still unclear, but some evidence linking SD, ER stress, and neuronal excitability comes from studies carried out in the hippocampus. Specifically, it has been demonstrated that Ca^2+^ depletion in the ER reduces neuronal intrinsic excitability and that this effect is mediated by an increased expression of the hyperpolarizing *h* channels in neuronal somas [50]. Similarly, SD led to a decline in cellular excitability with a mechanism mediated by the same *h* channels [51, 52]. Therefore, ER stress associated with SD could promote local sleep like phenomena by transiently modifying cellular excitability.

Consistently with the hypothesis that MAM number increases to sustain ATP production and increased cellular metabolic activity during SD, we found that flies genetically overexpressing MAMs are more active in normal baseline condition and have shorter sleep duration and less consolidated sleep than wild type controls. In principle, the higher number of MAMs may guarantee a greater support of Ca^2+^ and lipids to mitochondria and increase ATP production and the tolerance to cellular stress imposed by wake, thus decreasing the need for sleep. However, when challenged with SD, mutant flies showed a sleep rebound similar to wild type controls, not only in terms of total sleep but also in terms of number and duration of sleep episodes. Thus, additional mechanisms, other than an increase in MAMs, likely contribute to the homeostatic sleep regulation in this fly model.

Finally, we acknowledge that our study presents some limitations. The EM analysis is limited to neuronal somas, and we ignore whether other brain cells, which promptly respond to sleep loss, such as microglia or astrocytes [53–55], display similar changes in the ER and mitochondrial dynamics. For example, there is evidence showing that astrocytes react to ischemic stress by increasing mitochondrial clustering and promoting MAMs at the perivascular end-feet [56]. However, whether these changes in astrocytes also occur after other forms of stress such as sleep loss is unclear. Furthermore, our EM analysis did not consider other neuronal compartments such as dendrites or synapses, whose changes in structure and molecular composition have been linked to sleep and wake. Although direct evidence showing morphological changes of ER and mitochondria following sleep loss at synapses is missing, several proteomic analyses carried out in biochemical preparations enriched in synapses (synaptosomes) of sleep-deprived rats revealed that key proteins involved in protein folding, regulation of calcium homeostasis, and mitochondrial respiratory chain increased their expression after SD [57]. These data are consistent with our transcriptomic findings in forebrain homogenates. Therefore, it might be not surprising that rearrangements of ER and mitochondria similar to the ones observed in neuronal somas could also occur at the synaptic level as response to SD. Finally, in our study, the duration of SD was of 6 h for EM studies, while it was of 4 h for the transcriptomics studies. Although unlikely, we cannot exclude that a longer SD could highlight additional differentially expressed genes for ER and mitochondria, which could eventually unmask other ER and mitochondrial functions promoted or inhibited by sleep loss.

## Conclusions

SD reshapes the transcriptional networks of ER and mitochondria related genes and leads to the formation of MAMs in cortical neurons. MAMs are critical hot spots for the communication between ER and mitochondria thanks to their capacity to orchestrate the exchange of mediators necessary for granting a normal cellular function. Therefore, the increased number of MAMs associated with SD could be a compensating mechanism to re-establish cellular homeostasis. In addition, MAMs may play a role in the regulation of sleep, although the mechanisms linking MAMs to sleep homeostasis needs further investigation.

## Methods

### Mice experiments

#### Animals

Four-week-old C57BL/6J male mice were used in this study with the exception of gene expression analysis, in which we have referred to our previous database NCBI GEO GSE48369, obtained using adult (9–10 weeks old, of either sex) heterozygous 2′, 3′-cyclicnucleotide 3′-phosphodiesterase (CNP)-eGFP-L10a bacterial artificial chromosome (BAC) transgenic mice [19]. For western blot and EM experiments, mice were housed in recording boxes for the duration of the experiment (light/dark (LD) 12:12, light on at 8 am, 23 ± 1 °C; food and water available ad libitum and replaced daily at 8 am). All the animals were monitored with infrared cameras. To estimate sleep and wake, we used a custom-made algorithm to measure motion in these mice. We previously described that this method, albeit it cannot distinguish between NREM and REM sleep, reliably distinguishes sleep and wake with a concordance with electroencephalographic (EEG) recordings of about 90% [19, 58]. Analysis of motion as a proxy for sleep and wake detection prevented the implant of EEG electrodes that inevitability provokes inflammation and can alter subsequent morphological analysis. Sleep mice were euthanized during the light phase (at ~3.00–5.00 PM) following a long period of sleep (> 45 min, discontinued by period of wake of < 4 min) and after spending no less than 70% of the previous 6–7 h awake. For all mice, sleep deprivation was manually performed by an experimenter during the light phase by exposing the mice to novel objects and occasionally to a running wheel when animals appeared drowsy. Mice were never disturbed during eating or drinking. The duration of sleep deprivation was of 6 h for electron microscopy and western blotting studies and of 4 h for the previously performed transcriptomics experiments. All procedures involving animals adhered to the Animals (Scientific Procedures) Act 1986 and Amendment Regulations 2012 as outlined in UK law and approved by the University of Bristol Animal Welfare and Ethics Review Board.

#### Gene expression analysis

Firstly, from an analysis of the literature, we have identified a detailed catalog of the ER and mitochondrial proteins. ER proteins were obtained from mouse brain by way of multidimensional separation techniques in combination with high-resolution tandem mass spectrometry [59], while mitochondrial proteins were obtained using in-depth tandem mass spectrometry (MitoCarta2.0 [60]). For the ER, we have obtained the gene names from the names of ER proteins, and for the mitochondria, we have extracted the gene names from the MitoCarta2.0 database but only considering those relative to CNS. Doing so, we created a mouse brain ER (ER genes) and mitochondrial (Mito genes) gene lists.

Secondly, we used the array data available at NCBI GEO database ([19]; GSE48369) to perform gene expression analysis of forebrain samples collected from sleeping and sleep deprived mice. Samples of this database were collected using the genetically targeted translating ribosome affinity purification (TRAP) methodology from BAC transgenic mice expressing EGFP tagged ribosomal protein L10a in oligodendrocytes. BacTrap technique was used to isolate transcripts from oligodendrocytes (the immunoprecipitated portion, IP). However, the resulting non precipitated portion formed the unbound sample (UB) that was enriched in all the remaining cell types (neurons and other glia cells). RNA transcripts were extracted in IP and UB samples, but in the present study, we used array data obtained only from the UB samples of sleeping (S) and sleep deprived (SD) mice. Data were normalized within each behavioral state group using Robust Multiarray Average. To identify transcripts that were differentially expressed across S and SD, comparisons were carried out using the Welch’s t test with Benjamini and Hochberg false discovery rate (FDR) multiple-test correction. All the unique transcripts with a fold change > 30% and *p* < 0.01 were considered as genes upregulated in SD, while unique transcripts with a fold change < 30% and *p* < 0.01 were considered as genes downregulated in SD. To identify ER and Mito genes that were differentially expressed in upregulated and downregulated SD genes, we intersected the ER and Mito genes with upregulated and downregulated SD genes. Finally, protein to protein interaction (PPI) networks were created and analyzed using STRING V11 [61].

#### Western blotting

*S* (*n* = 5) and *SD* (*n* = 5) mice were euthanized with cervical dislocation and then decapitated; the whole brain was rapidly collected, dissected in cerebellum and forebrain, frozen on dry ice, and maintained at – 80 °C for further use. Through a glass/glass tissue homogenizer, forebrain samples were homogenized in freezing homogenization buffer containing 10 mM HEPES, 1.0 mM EDTA (Sigma), 2.0 mM EGTA (Promega), 0.5 mM DTT (Bioston BioProducts), 0.1 mM PMSF (Invitrogen), Protease Inhibitor Cocktail (Fluka), 100 nM microcystin (Roche). Next, 100 μL of each sample was boiled in 10 μL of Sodium Dodecyl Sulfate (SDS) at 90/95 °C for 10 min and kept at – 80 °C. The protein concentration was evaluated with bicinchoninic acid assay.

Equal amounts of protein from each animal (3 μg for VDAC1 and 6 μg for STIM2) were loaded onto the same gels with sample loading order randomized. Homogenate samples of each S and SD mice were resolved by Tris-HCl gel electrophoresis in 1X Tris/Glycine/SDS running buffer. Then, the proteins were transferred onto 0.45 μm pore size nitrocellulose membranes in 1X Tris/Glycine/Methanol transfer buffer. After transfer, membranes were stained with Ponceau S, acquired for total protein quantification at the Chemidoc (Bio-Rad), and then washed 3 times with 1X PBS 0.1% Tween-20. The membranes were immunoblotted as follows. First, they were blocked in 5% non-fat dry milk in 1X PBS 0.1% Tween-20 with gentle shaking for 1h at room temperature; then, they were incubated for 2 h at room temperature with gentle shaking and overnight at 4 °C with one of the following primary antibodies: anti-voltage-dependent anion channel 1 (VDAC1, Abcam #15895, 1:2000) or Stromal Interaction Molecule 2 (STIM2, Cell Signaling #4917, 1:1000) diluted in 1X PBS 0.1% Tween-20 with 0.5% non-fat dry milk. Next, membranes were rinsed 3 times in 1X PBS 0.1% Tween-20 and then incubated with goat anti-rabbit secondary antibody (1:5000) diluted in 1X PBS 0.1% Tween-20 with 3% non-fat dry milk for 90 min at room temperature with mild oscillation. Membranes were washed 3 times in 1X PBS 0.1% Tween-20 and, in the end, with ddH_2_O, incubated with enhanced ECL Chemiluminescence Reagent (ECL-Prime, GE Healthcare), and the bands were revealed by exposition to Molecular Imager Chemidoc XRS+ (Bio-Rad). The optical density of the bands was quantified by the ImageJ software (National Institutes of Health). Since housekeeping proteins (e.g., α-actin and β-tubulin) can be affected by sleep and wake, they were not used as internal standard. Instead, optical density values were normalized to total protein loading obtained by the ponceau S staining [62] (normalized experimental signal = observed experimental signal/lane normalization factor; the lane normalization factor = observed signal of total protein for each lane/by the highest observed signal of total protein on the blot). The nonparametric Mann-Whitney test was used to compare *S* and *SD* data sets. Alpha was set to 0.05.

#### Electron microscopy

*S* (*n* = 4) and *SD* (*n* = 4) mice were perfused intracardially under deep anesthesia with a solution of 0.05 M phosphate buffered saline followed by 2.5 % glutaraldehyde and 4% paraformaldehyde dissolved in 0.1 M sodium cacodylate buffer (41°C and pH 7.4). Brains were removed and kept in the same fixative overnight at 4 °C. Brain slices were cut on a vibratome and kept in a cryoprotectant solution until the day of processing. Sections were rinsed 3 × 10 min each in cacodylate buffer and incubated for 1 h on ice with a solution of 1.5 % potassium ferrocyanide/2 % osmium tetroxide. After three rinses in ddH_2_O, they were exposed to a solution of 1 % thiocarbohydrazide for 20 min at room temperature. Sections were washed with ddH_2_O and placed in 2% osmium tetroxide for 30 min, washed again, and incubated overnight with 1% uranyl acetate at 4 °C. The following day, the tissue was stained with a solution of lead aspartate, dehydrated, and embedded with Durcupan resin and ACLARfilm. Small squares of tissue from frontal cortex neurons (layer II-III) were glued on the tip of a metal pin and coated with silver paint to minimize specimen charging during imaging.

#### Image acquisition and analysis

Images were obtained using serial block-face electron microscope Zeiss Gemini SEM 450 (Carl Zeiss NTS Ltd) equipped with 3View® technology (Gatan Inc.) and a backscattered electron detector. Images were acquired using an aperture of 30 μm, high vacuum, acceleration voltage of 2 kV; image resolution (xy plane) was between 1 and 3 nm. At low magnification, pyramidal neurons were sampled in layer II of the frontal cortex using previously established criteria [63–65]: their triangular shape, large nucleus with 0 or 1 indentation, and only a thin rim of cytoplasm, and emergence of apical or basal dendrites. Next, high magnification images of the cytoplasmatic area of neuronal somas were collected. Images were processed and analyzed using imaging software (FIJI). We sampled 139 neurons from S and 132 neurons from SD mice. MAMs were manually identified by measuring the distance between the ER and the mitochondria, which typically was between 10 and 20 nm. Pseudo contacts with distance above 20 nm were not considered MAMs. Moreover, we measured the length of the tracts in which the ER and mitochondrial membranes were opposed to each other. When ER cisternae contacted the same mitochondria at multiple sites, the contacts were annotated as multiple MAMs. Using the same criteria, we measured number and length of the contacts between ER and plasma membrane (PAM) and contacts between mitochondria and plasma membrane (Mito-PM). Finally, we measured the ability of mitochondria to form clusters by counting the number of mitochondria that were in contact with each other (by being within 20nm of each other). Unpaired *t*-test was used to compare distributions of S and SD data sets. Alpha was set to 0.05.

### Drosophila experiments

Fly stocks and crosses were maintained and tested on standard *Drosophila* medium at 25 °C, 70% relative humidity with a 12 h:12 h LD cycle. For the experimental line *UAS-linker* flies [26] were crossed to the pan-neuronal driver line *elav-GAL4* (BL8760; Bloomington *Drosophila* Stock Center, IN, USA), and controls were generated by crossing Gal4 promoter and UAS responder lines to *Canton-S w-* (Dr Scott Waddell, University of Oxford, UK). Individual male progeny flies were collected between 2 and 4 days post eclosion and loaded in the *Drosophila* Activity Monitor system (DAM2, Trikinetics Inc., Waltham, MA, USA) placed inside a light- and temperature-controlled incubator. Locomotor data was collected in 1 min bins, and sleep was defined as five or more minutes of inactivity [66–68]. Sleep parameters were quantified in MATLAB using the SCAMP script. Baseline activity and sleep measurements were averaged over the 4 days of LD and further split into the day (i.e., when lights were on) and night (i.e., lights off) components. Sleep deprivation was performed by placing the DAM monitors for both control and experimental flies on a shaker controlled by a timer. Flies were sleep deprived for the whole 12 h night phase of day 5 by activating the shaker for 5 s in every 1 min and followed by a day of recovery. Flies that had died before the end of the experiment were removed from the analysis. Kruskal-Wallis followed by Dunn’s multiple comparisons test was used to compare sleep/wake data from transgenic and controls. Sleep stability was calculated by subtracting the maximum sleep bout duration from the maximum wake bout duration. Positive values indicate a more wakeful state while negative values are indicative of a more stable sleep state. To test the effect of genotype on the sleep rebound after SD, we computed the difference in sleep time between *SD* and *S* and compared values between groups using a Kruskal-Wallis’s test. Alpha was set to 0.05 and opportunely corrected when multiple comparisons were performed (Bonferroni’s correction).

## Supplementary Information

**Additional file 1: Fig. S1.** Network analysis predicting protein-protein interaction for ER genes that are upregulated in SD. In this and in all subsequent figures, the network nodes are proteins, while the edges represent the predicted functional associations. Line thickness indicates strength of the association (edge confidence - low (0.150); medium (0.4); high (0.7); highest (0.9) [61].. **Fig. S2.** Network analysis predicting protein-protein interaction performed with for ER genes that are downregulated in SD. **Fig. S3.** Network analysis predicting protein-protein interaction for Mito genes that are upregulated in SD. **Fig. S4.** Network analysis predicting protein-protein interaction for mito genes that are downregulated in SD.

**Additional file 2.** Functional enrichment of ER genes not affected by SD (top 50 biological processes with strength of enrichment >1).

**Additional file 3.** Functional enrichment of Mito genes not affected by SD (top 50 biological processes with strength of enrichment >1).

**Additional file 4 **Western blots: **Fig. S1.** STIM2 gel. **Fig. S2.** STIM2 Ponceau S. **Fig. S3.** VDAC1 gel. **Fig. S4.** VDAC1 Ponceau S.

**Additional file 5. **Quantification of western blot data for S (*n* = 5) and SD (*n* = 5) mice.

## Data Availability

All data generated or analyzed during this study are included in this published article, its supplementary information files, and publicly available repositories. The transcriptome dataset analyzed during the current study is available at NCBI GEO database ([19]; GSE48369). The EM dataset is available at [69] DOI: 10.6084/m9.figshare.21671189, while the *Drosophila* dataset is available at [70] DOI: 10.6084/m9.figshare.21656402. Western blot data are included in Additional files 4 and 5.
