# Supplementary figures and images for: Increased interaction between endoplasmic reticulum and mitochondria following sleep deprivation
Source: BMC Biol. 2023 Jan 4;21:1. doi: 10.1186/s12915-022-01498-7 (PMC9814192; doi:10.1186/s12915-022-01498-7)

**Additional file 1**

Figure S1


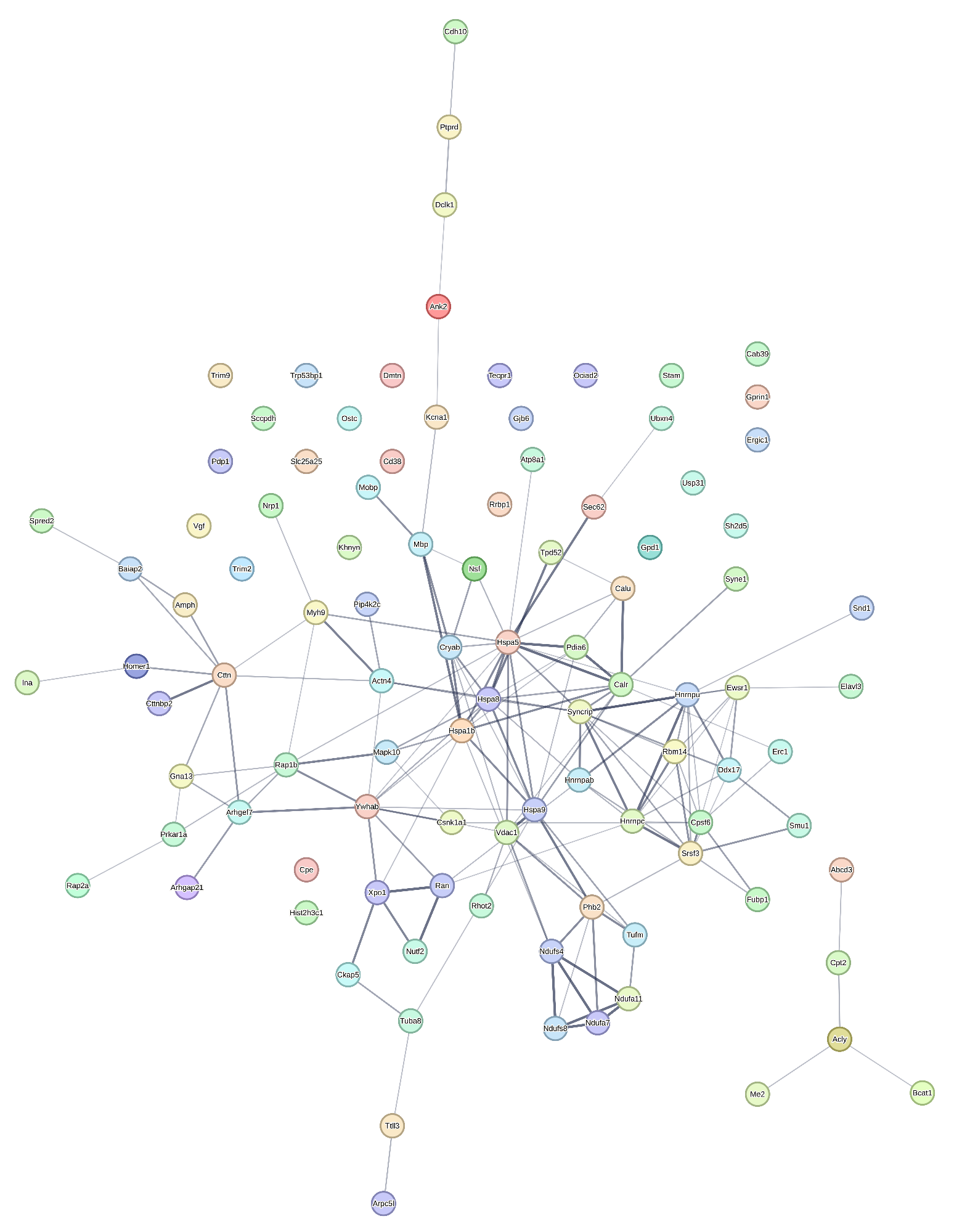


Figure S2


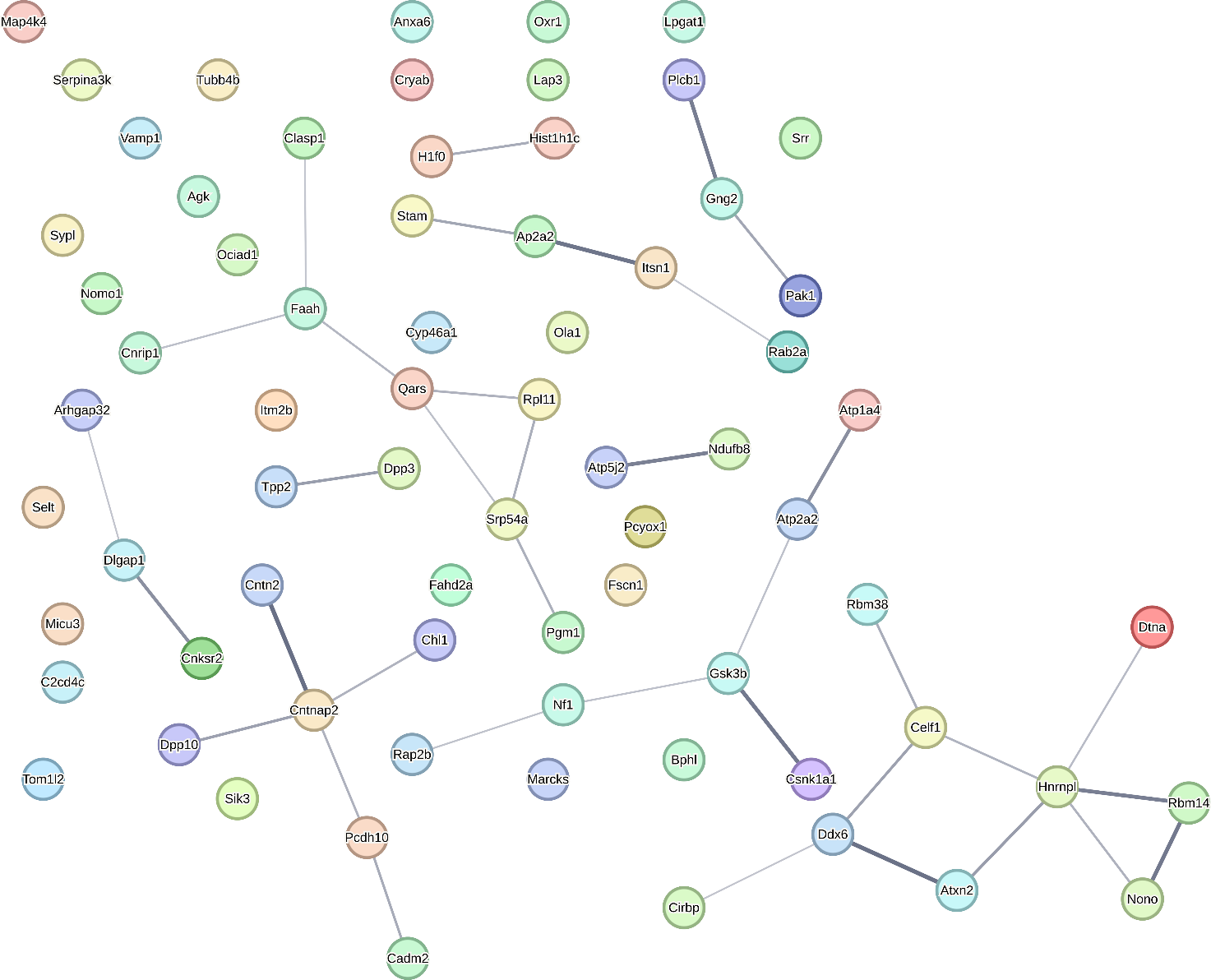


Figure S3


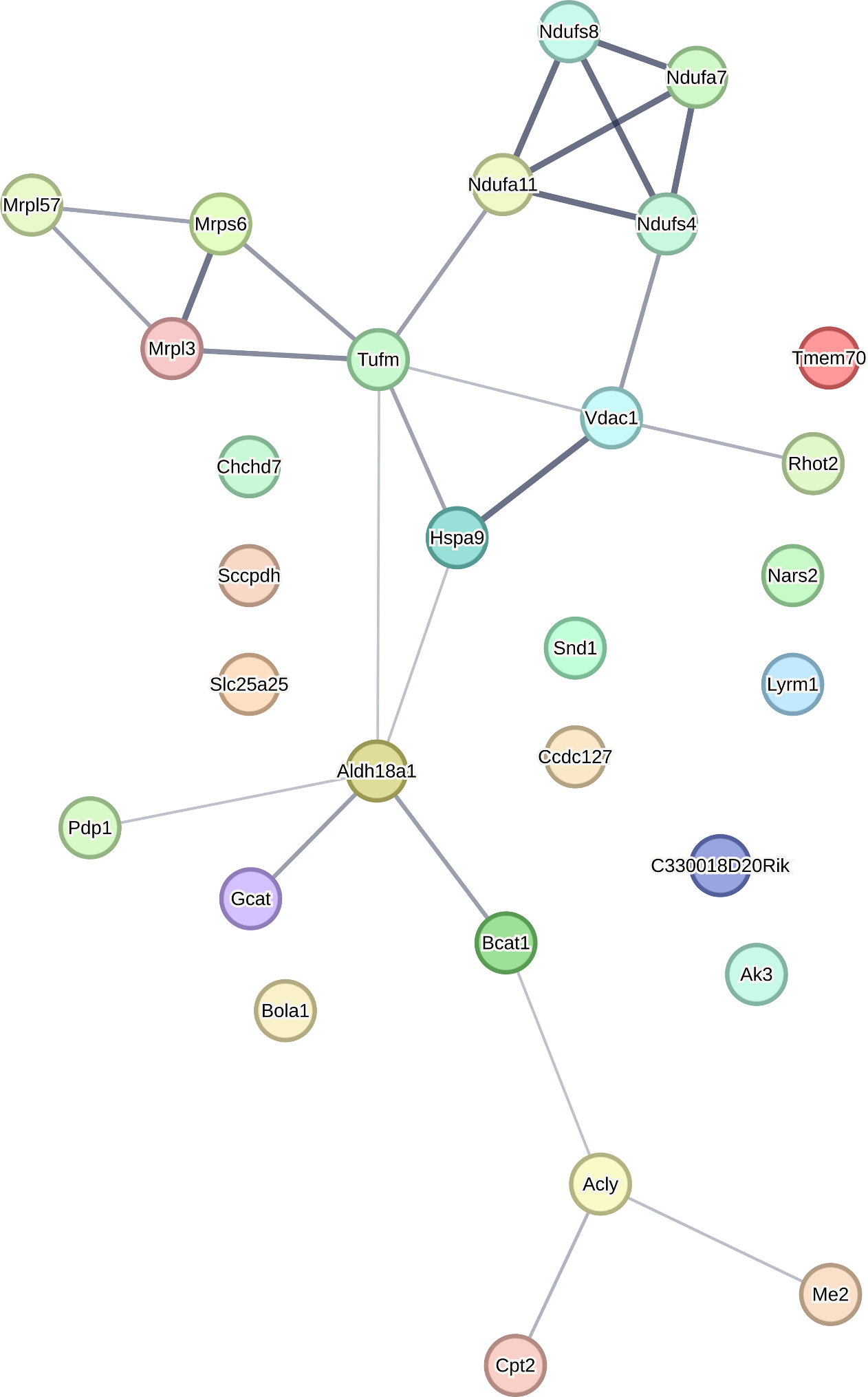


Figure S4


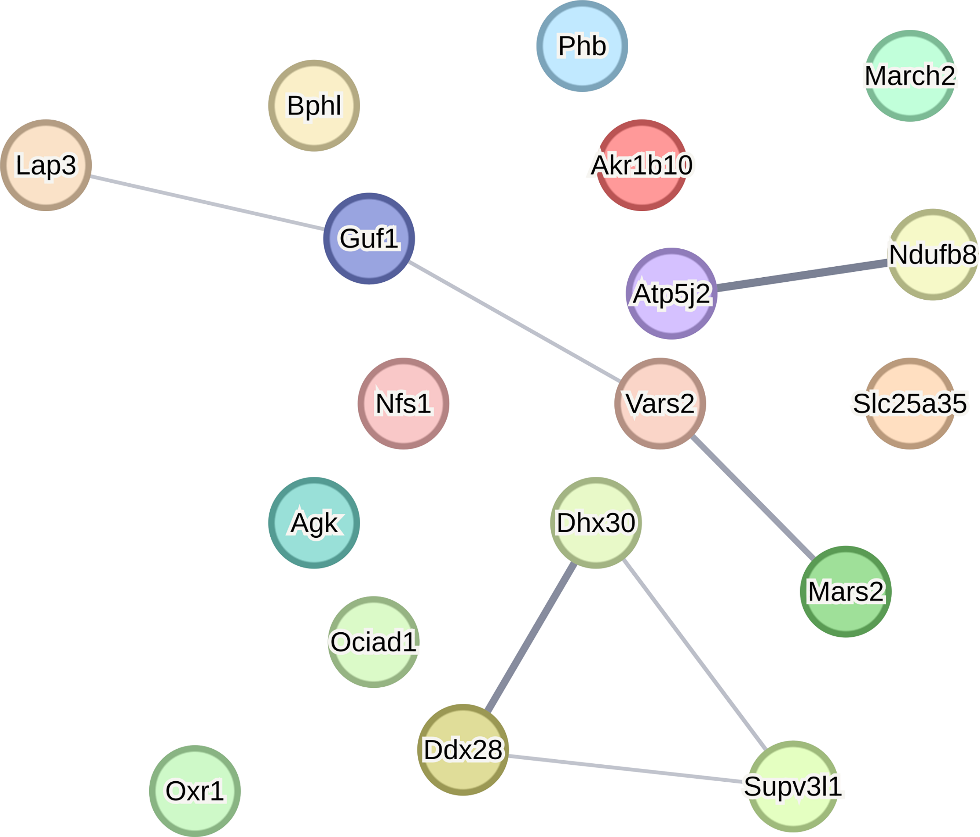

Supplement: Supplementary file 1 — Additional file 1: Fig. S1. Network analysis predicting protein-protein interaction for ER genes that are upregulated in SD. In this and in all subsequent figures, the network nodes are proteins, while the edges represent the predicted functional associations. Line thickness indicates strength of the association (edge confidence - low (0.150); medium (0.4); high (0.7); highest (0.9) [61].. Fig. S2. Network analysis predicting protein-protein interaction performed with for ER genes that are downregulated in SD. Fig. S3. Network analysis predicting protein-protein interaction for Mito genes that are upregulated in SD. Fig. S4. Network analysis predicting protein-protein interaction for mito genes that are downregulated in SD. [file 12915_2022_1498_MOESM1_ESM.docx]

**Additional file 4**

Figure S1


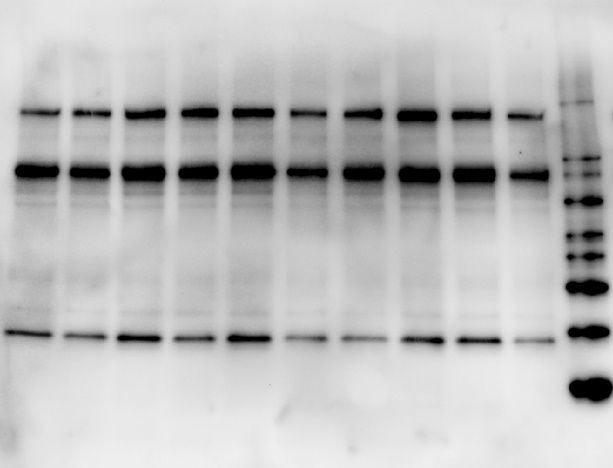


Figure S2





Figure S3


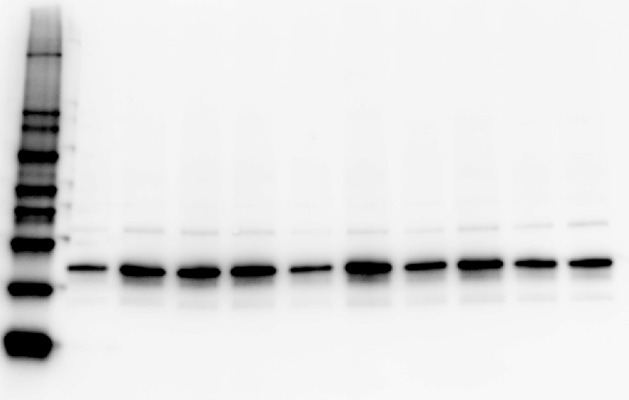


Figure S4

Supplement: Supplementary file 4 — Additional file 4 Western blots: Fig. S1. STIM2 gel. Fig. S2. STIM2 Ponceau S. Fig. S3. VDAC1 gel. Fig. S4. VDAC1 Ponceau S. [file 12915_2022_1498_MOESM4_ESM.docx]
